# The myriad possibility of kidney organoids

**DOI:** 10.1097/MNH.0000000000000498

**Published:** 2019-03-19

**Authors:** Pinyuan Tian, Rachel Lennon

**Affiliations:** a Wellcome Centre for Cell-Matrix Research, Faculty of Biology, Medicine and Health, University of Manchester; b Department of Paediatric Nephrology, Manchester University Hospitals NHS Foundation Trust, Manchester, UK

**Keywords:** differentiation protocol, drug screening, induced pluripotent stem cells, kidney disease modelling, kidney organoids, kidney-on-a-chip, nephrogenesis

## Abstract

**Purpose of review:**

Human kidney development and the mechanisms of many kidney diseases are incompletely understood partly due to the lack of appropriate models. Kidney organoids derived from human pluripotent stem cells (hPSCs) are a new and rapidly developing in-vitro system covering the window of early nephrogenesis and having the capacity for disease modelling. The application of global analytic tools such as RNA sequencing and proteomics is providing new and unexpected insights into kidney organoids with relevance for development and disease. In this review, we focus on the most significant advances in the field over the last 2 years.

**Recent findings:**

There have been several protocol modifications for the differentiation of hPSCs into kidney organoids, including the additional step of implantation into mice. These changes have improved the vascularization and maturity of the major cell types in the organoids, increased the production scale, and reduced the cost and labour intensity of culturing organoids. Single-cell RNA sequencing and global proteomics of kidney organoids have provided important insights into the multiple cell populations, origin of cells, and regulatory relationships between genes. There has been an increase in research using patient-derived induced pluripotent stem cells (iPSCs), or combining gene editing with iPSC-derived kidney organoids as a novel disease-modelling platform for improving our understanding of disease mechanisms, drug testing and discovery, and the potential for personalized therapy. Finally, there has been progress in culturing hPSCs-derived kidney cells in microfluidic kidney-on-a-chip devices and this may provide a means of further improving the maturity of kidney organoids.

**Summary:**

The review summarizes the latest progress on kidney organoids including differentiation protocols, analysis tools, and applications. Despite some limitations, hPSC-derived kidney organoids are authentic and practical models for investigating kidney development and disease and progressing understanding about tissue regeneration, drug screening, and disease modelling.

## INTRODUCTION

The creation of kidney structures from human pluripotent stem cells (hPSCs) has been extensively studied in the past 5 years. It is one of the most attractive solutions for disease modelling, drug screening, and perhaps even renal replacement. hPSCs have an unlimited capacity for self-renewal and with the appropriate conditions they differentiate into de novo nephron structures [1]. In particular, induced pluripotent stem cells (iPSCs) reprogrammed from patient-derived somatic cells (e.g., dermal fibroblasts, or peripheral blood cells) enable the development of patient-specific, immuno-compatible tissues, which provide ideal in-vitro disease models for understanding underlying mechanisms and potential treatment [2]. The generation of iPSC lines requires expertise and the methods are time consuming therefore iPSC resources such as the human iPSCs initiative based at the Sanger Institute in Cambridge [3] provide important platforms for researchers to study human iPSC lines generated from patients with a range of inherited genetic diseases as well as control lines from healthy donors.

In addition to the direct differentiation of PSCs into kidney lineage, nephron progenitor cells with the capacity to form kidney-like tissues, many protocols have induced the differentiation of PSCs to generate three dimensional structures called kidney organoids, which contain multiple renal cell types and are capable of self-organization. These organoid structures are considered a realistic and practical approach for investigating and understanding human kidney development and disease [4,5]. Several recent reviews have summarized and compared the current directed differentiation protocols for generating kidney organoids from PSCs [1,2,6–9] and these are broadly based on inducing differentiation by the coordinated introduction of growth factors and chemical regulators (Table 1). Here we focus on the latest findings in the organoid field, including recent changes in protocols and also detail the potential applications of kidney organoids in disease modelling and regenerative medicine. 

**Box 1:**
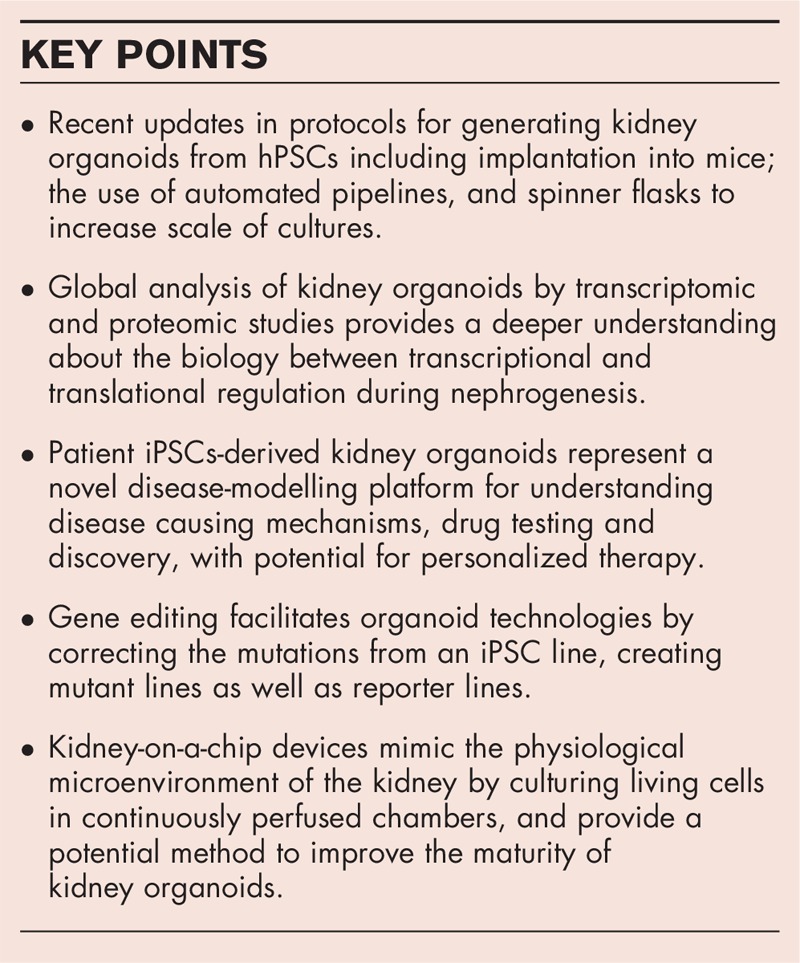
no caption available

## PROTOCOLS FOR GENERATING KIDNEY ORGANOIDS FROM HUMAN PLURIPOTENT STEM CELLS

Although the organoids derived from hPSC contain multiple renal cells and complex kidney-like structures, they have a number of limitations [2,6]. First, the glomeruli, proximal, and distal tubules, as well as the collecting duct structures in the organoids are not fully mature even after prolonged culture, and they lack vascularization. Second, the size of the organoids is far smaller than a normal human foetal kidney and if they continue to expand in culture, hypoxia and metabolic deficits have been observed [10]. Third, from a practical perspective the differentiation processes are labour-intensive, time-consuming, and high-cost. Finally, there are significant variations between cell lines and differentiation batches. Especially for iPSCs, significant line-to-line and batch to batch variations are observed even when the differentiation conditions have been optimized for each iPSCs line [11,12,13^▪▪^,^14^^▪▪^].

To address these limitations, a number of research groups working on kidney organoids have reported modifications in differentiation protocols in the past 18 months [10,12,13^▪▪^,^15^,^16^]. A set of defined serum-free culture conditions suitable for the maintenance and growth of kidney organoids was identified to facilitate potential clinical translation [17]. Two groups following the same differentiation protocol implanted the hPSC-derived in-vitro kidney organoids either under the skin [18^▪^] or beneath the kidney capsule [10] of immunodeficient mice. In these studies, functional vascularization was observed in the organoids, as well as the increased maturity of the nephron structures including: mature components of the glomerular basement membrane (GBM), fenestrated endothelial cells, and podocyte foot processes. These investigations have demonstrated that in-vivo implantation is an encouraging approach for vascularization, and can extend organoid development further. In addition, Nishinakamura's group reported a modified protocol capable of reconstituting higher order organ structures containing the dichotomously branching ureteric tree in an organoid, which would be essential for expansion of the organoid size and drainage of filtrate and beneficial for organ-scale tissue reconstruction [16].

Scaling up the number of kidney organoids in culture has involved the use of 96-well and 384-well plates [12,13^▪▪^,^19^]. A recent preprint article reported the use of a robotic plating system to enable the production of large numbers of highly reproducible organoids by comparison with manual plating [15]. The authors showed that kidney organoids plated by a three dimensional bioprinter showed equivalent morphology, component cell types and gene expression compared with those previously reported by manual generation [15,19]. In an impressive further development, Freedman's group established the first fully automated, high throughput, robotic pipeline to manufacture and analyse kidney organoids in microwell arrays. This system appears to be suitable for high-throughput screening including all steps from plating, to differentiation, to analysis and imaging [13^▪▪^].

To address the issue of cost and scale, an alternative, simple and inexpensive method to grow kidney organoids in bulk from iPSCs was reported using spinner flasks [20^▪^]. Large numbers of embryoid bodies could be induced into organoids of equivalent quality in spinner flasks in ‘KnockOut Serum Replacement’ medium from three different iPSC lines without line-specific optimization. For the issue of variation in kidney organoid cultures, Phipson *et al.* studies the factors associated with variation and reported that the greatest source of variation was from technical parameters rather than the cell line. From these findings it would seem necessary to perform differentiations between comparison lines concurrently to mitigate the effects of technical factors in the variation [14^▪▪^].

## SINGLE-CELL RNA SEQUENCING AND PROTEOMIC ANALYSES OF KIDNEY ORGANOIDS

Despite the latest progress with differentiation protocols, kidney organoids are still far from a human kidney or a transplantable kidney regarding the size, scale, maturity, and functions. To further improve differentiation strategies, it is necessary to increase knowledge about the development of the cells within these organoids [21^▪▪^].

RNA sequencing (RNA-seq) analysis, especially single cell RNA-seq (scRNA-seq) or single nucleus RNA-seq (snRNA-seq), are emerging tools for revealing complex cell populations, uncovering regulatory relationships between genes, and for tracking the trajectories of distinct cell lineages during development [22]. Two comprehensive molecular maps describing the cell diversity in kidney organoids were generated based on two distinct differentiation protocols. These scRNA-seq and snRNA-seq results demonstrate that organoids generated from both protocols are relatively similar, despite the use of different culture media and conditions during differentiation [8]. First, they contain at least 12 separate kidney cell types including podocytes, proximal tubular cells, Loop of Henle cells, and endothelial cells. Second, both differentiation protocols showed some off-target, nonrenal cell types such as muscle cells, and neurons. This consequence could be dramatically reduced by inhibiting the receptor NTRK2, which is the cognate receptor of brain-derived neurotrophic factor [21^▪▪^]. Moreover, snRNA-seq data indicated that kidney organoid cells are relatively immature compared with either foetal or adult human kidney cells [6,13^▪▪^,^21^^▪▪^]. Another report claimed that their organoids contain at least four different mature cell types (podocytes, proximal tubules, distal tubules, and endothelial cells) but could only detect two mature cell types using scRNA-seq possibly due to low cell abundance, lack of specific markers, and technical difficulties in getting cells into single-cell suspension for fluorescence-activated cell sorting [23].

Lineage-tracing using the single cell transcriptome of day 18 and 25 organoids demonstrated that *SIX2* marks several distinct cell types, including a muscle-like population, renal stroma, and a putative nephron progenitor cell population, which contributes to nephron formation but not to the branching ureteric epithelium [15]. Comparisons of the cellular transcriptomes of mouse and human kidney [24], human adult and fatal kidney, normal and tumour kidney [25] have also been published in parallel and have highlighted differences in nephron-forming programs and defined the cellular identity of normal and cancerous human kidney cells. For example, scRNA-seq analysis of both human foetal kidney and kidney organoids derived from genetically engineered human iPSCs shows substantial overlap between nephron progenitor cells and the interstitial progenitor cells, whereas mouse kidney has a strict lineage boundary between these cell populations [15,24]. In another study, childhood Wilms tumour cells were found to match the cellular identity of specific foetal cell types (ureteric bud and primitive vesicle cells) based on gene expression and similarity analysis, which suggests that Wilms tumour cells are aberrant fetal cells [25].

**FIGURE 1:**
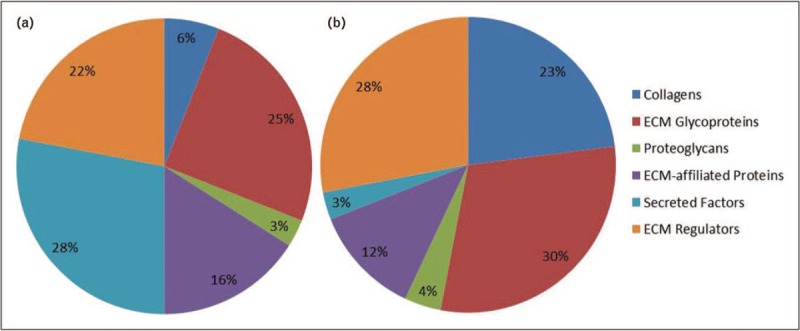
Transcriptomic and proteomic analysis of the matrisome in induced pluripotent stem cell-derived kidney organoids by RNA sequencing (a) and mass spectrometry (b). Gene ontology classification of the matrix genes (a) and proteins (b) detected in organoid glomeruli. Matrisome components do not always show a direct correspondence between quantified gene expression and protein abundance and this appears to be the case with the organoid glomeruli matrix. This could be explained by structural proteins such as collagens having long half-lives and slow turnover while secreted factors are less consistently detected in matrix proteomic studies.

Although transcriptomic analysis using RNA-seq allows the quantification of gene expression, mass spectrometry-based proteomics enables the examination of global protein expression, and improved understanding about the biology between transcriptional, translational and posttranslational regulation. Hale *et al.* reported a comprehensive transcriptional and proteomic study of human iPSC-derived kidney organoids. The authors demonstrated that organoid glomeruli (OrgGloms) represent a novel in-vitro model of human glomeruli by showing enhanced podocyte-specific gene expression, polarized protein localization and mature GBM components. These included laminin-α5β2γ1 (LAM-521), collagen IV α1/2/3/5/6, nidogen 1and 2, collagen XVIII, agrin and perlecan and the findings are were compared with a conditionally immortalized human podocyte cell line cultured in two dimensional [26^▪^]. Significantly, there were differences between the transcript and protein abundance for matrix components (Fig. 1) and this may reflect different turnover rates in matrix proteins. The low protein abundance of collagen IV α3 in OrgGloms may reflect the absence of vascularization or flow and in support of this hypothesis collagen IV α3 was detected using chain-specific antibodies in glomerular structures that formed after implanting hPSC-derived kidney progenitors into immunodeficient mice [18^▪^]. Previous studies have shown that laminin and collagen IV isoforms switch during glomerular maturation from LAM-111 to LAM-511 to LAM-521, collagen IVα112 to collagen IVα345, respectively. However, the molecular or environmental cues for these switches are not well understood. Although the full collagen IV network of α345 was not detected in OrgGloms possibly due to incomplete maturation, low protein abundance, or analytical approach, there appears to be a sufficient window of maturation in kidney organoid differentiation to allow further investigation of GBM assembly and extracellular matrix deposition during nephrogenesis.

## KIDNEY DISEASE MODELLING AND GENE EDITING

Patient-derived iPSCs kidney cells represent a novel disease-modelling platform for understanding mechanism, drug testing and discovery, and examining the potential for personalized therapy. A number of genetic kidney diseases, especially those with limited access to human diseased tissue have been selected for modelling using patient-derived iPSCs. For example, Alport syndrome patient-derived podocyte-like cells were shown to exhibit dysfunctional integrin signalling and potassium channel function, which may associate with the podocyte loss and dysfunction seen in Alport syndrome [27]. Furthermore, OrgGloms generated from a patient with congenital nephrotic syndrome due to a mutation in *NPHS1* displayed reduced levels of both nephrin and podocin. In addition the bulk production of more than 3000 OrgGloms in a single differentiation enabled screening for podocyte toxicity [26^▪^].

Due to the genetic variation between patients the interpretation of transcriptomic and proteomic data between iPSC lines will be challenging. In addition to the analysis of unaffected family member control lines, many researchers are now focusing on generating genetically engineered isogenic controls using the state-of-art gene editing tools, such as clustered regularly interspaced short palindromic repeats/associated systems 9 [(CRISPR)/Cas9], which provide a powerful and unique approach to study human kidney development and disease [7,28,29]. For example, iPSCs derived from a patient with nephronophthisis-related ciliopathy due to mutations in *IFT140* and isogenic gene-corrected iPSCs were differentiated to kidney organoids [30]. As anticipated, uncorrected organoids had shortened tubules and club-shaped primary cilia, whereas the gene edited correction rescued this phenotype. Similarly, genetic correction of the single amino acid mutation of iPSCs generated from a congenital nephrotic syndrome patient containing *NPHS1* mutations has restored nephrin localization and phosphorylation, colocalization with NEPH1 and podocin, and slit diaphragm formation [31].

Gene editing methods have utility for correcting the pathogenic mutation in an iPSC line, but also for creating mutant lines. Rather than using patient-derived cells, Freedman's group generated a CRISPR-mutant *PKD1* and *PKD2* knockout hPSCs lines, and derived kidney organoids using their robotic pipeline to recapitulate some characteristic features of polycystic kidney disease (PKD) *in vitro*. High-throughput screening revealed an unexpected role for myosin in PKD [13^▪▪^]. They also demonstrated a role for biophysical properties and adherent forces, which appear to play a critical role in increasing or decreasing cyst formation [11]. The same group also established CRISPR/Cas9-podocalyxin-knock-out hPSCs, and derived the engineered hPSCs into podocytes to demonstrate the conserved and essential role of podocalyxin in mammalian podocyte maturation [32]. This promising start suggests that considerable further information about disease mechanisms will emerge from extended research on the patient and corrected cell lines.

Another application of gene editing is the generation of reporter lines. Three kidney-specific reporter cell lines, SIX2-GFP, NPHS1-GFPand SIX2-GFP/NPHS1-mKate, have been created to allow live cell monitoring of organoid differentiation. The maturation of kidney progenitors and podocytes can be monitored in live cells engineered by CRISPR/Cas9-mediated fluorescent tagging of kidney lineage markers (SIX2 and NPHS1). These reporter lines are also of benefit for optimizing the procedures suitable for drug discovery [23]. In another study, SIX2 knock-in iPSC-derived kidney organoids were generated by combining reprogramming, CRISPR/Cas9 gene-editing and organoid technologies to monitor nephron progenitor cells during kidney organoid differentiation and to study human nephron lineage relationships *in vitro*[15].

## KIDNEY-ON-A-CHIP

The lack of organoid vascularization is one of the main drawbacks of the current differentiation protocols for kidney organoids. However, by implanting differentiating hPSCs into immunodeficient mice there was evidence of a blood supply from the mouse host in the glomerular structures that formed within the organoids [10,18^▪^]. Furthermore, a low level of ultrafiltration into nephron tubules was observed [18^▪^]. Although the mechanism behind this apparent further maturation has not been resolved, it seems likely that blood flow is necessary for later stage nephron formation and maturation.

Kidney-on-a-chip technology combines cell culture with microfluidic techniques, and therefore attempts to mimic the physiological microenvironments of the kidney by culturing living cells in continuously perfused chambers that recapitulate nephron and kidney-level functions. Combining with hPSCs differentiation approaches, this technology provides a potential method for further differentiation of kidney organoids.

Most of the current kidney-on-a-chip studies have used primary [33,34] or immortalized kidney cells including podocytes, endothelial cells, and tubular epithelial cell to form a functional glomerulus. However, since podocytes are highly specialized cells, primary podocytes only replicate for a short time, while immortalized podocytes appear to have lower suitability for toxicological screening [27]. hPSC-derived podocytes have been considered as an alternative source of cells, although it is necessary to determine the developmental stage of hPSCs-derived podocytes and their ability to recapitulate disease phenotypes [32].

Musah *et al.* reported an efficient (over 90%) and chemically defined method for directing iPSCs into mature podocytes (nephrin^+^, WT1^+^, podocin^+^, PAX2^−^) with evidence of cellular processes that may represent primary and secondary podocyte foot processes. After co-culturing the iPSCs-derived podocytes with primary human glomerular endothelial cells in a kidney-on-a-chip microfluidic device the authors describe the presence of extracellular type IV collagen [35]. Although intriguing, it is unclear from this study whether the cells are secreting the α112 network or the mature 345 network of type IV collagen which is an integral component of the mature GBM. The use of chain-specific antibodies for type IV collagen could help to resolve this issue and it would also be of interest to know whether other mature GBM components including laminin 521 are also secreted and assembled in this co-culture system. Overall this study supports the concept that fluid flow and stretch of the capillary wall may be required to achieve greater maturation of kidney structures but more investigation is required to determine the stage of maturity in these culture systems where direct contact between cell types is absent.

## CONCLUSION

**FIGURE 2:**
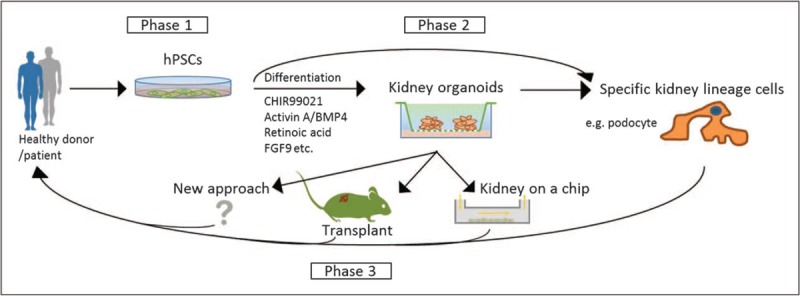
Kidney organoids derived from human pluripotent stem cells and the potential for renal regeneration. Phase 1, human pluripotent stem cells, including human embryonic stem cells derived from donor embryos and human-induced pluripotent stem cells generated from patients with kidney diseases, are promising candidates for renal regeneration. Phase 2, induction of human pluripotent stem cells differentiation towards the kidney lineage to generate three dimensional kidney-like organoids, or direct differentiation of human pluripotent stem cells (following genetic correction) into specific kidney lineage cells such as podocytes. Phase 3, after further methods development such as transplantation and the use of flow systems, the mature renal cells may be transferred back to patients as therapy to delay the progression of disease and potentially to improve kidney function.

Generating kidney organoids from PSCs has attracted extensive attention due to the promising potential for regenerative medicine and drug discovery. Unlike conventional cell culture, PSC-derived kidney organoids are self-organized to recapitulate kidney development *in vitro*, and they possess the architecture and geometric features of renal tissue seen *in vivo*. Compared with animal models, kidney organoids generated from human stem cells overcome the issue of species differences. Moreover, patient-derived iPSC organoids hold great promise in personalized disease modelling and drug testing, and provide hope for the development of gene therapies for inherited and acquired kidney diseases (Fig. 2).

Since hPSCs have the ability to differentiate into almost any cell type in the body, the organoid approach has been widely expanded to many other research fields such as heart [36], brain, retina, intestine and liver [37,38], and there has been significant scientific progress. For example, transplanted brain organoids in the rodent brain shows progressive neuronal differentiation and maturation, integration of microglia, and growth of axons to multiple regions of the host brain [39].

As with every model, kidney organoids from hPSCs have their limitations. Although studies are continually striving for improvement, the organoids produced by current protocols are not comparable with a human kidney in size, scale, maturity, and functions. Currently, researchers are seeking elusive clues for further differentiation. New culture conditions have been established, novel inhibitors have been identified using comprehensive analysis methods such as RNA-seq and proteomics, state-of-art tools including CRISPR/Cas9 have widely used to generate powerful genetic engineered cell lines. Mechanical force in the form of fluid flow could be one of the underlying factors, and microfluidic devices may enhance nephrogenesis.

Regardless, the current immature organoids represent an early development stage of nephron formation that have features in parallel with early human foetal kidney development. This stage of development has not been extensively studied due to limited access to foetal tissues, and therefore organoids provide a valuable platform to extend our knowledge on. For example, with limited understanding we consider that GBM is formed and matured from the 5th week of gestation in humans. Together with proteomic analysis, kidney organoids derived from hPSCs would help us to create a detailed picture of GBM assembly and early regulation. In addition, by the use of organoids derived from patients with inherited genetic diseases in GBM components, we have the opportunity to explore disease mechanisms and the potential to identify novel therapies. Thus despite some limitations hPSCs-derived kidney organoids appear to be realistic and practical models for understanding human kidney development and the early stages of inherited kidney disease.

## Acknowledgements

*We thank Dr Craig Lawless (Wellcome Centre for Cell-Matrix Research) for bioinformatic assistance withFig. 1, Professor Sue Kimber (Division of Cell Matrix Biology & Regenerative Medicine, University of Manchester) for critical review of the article and Joseph Luckman (Wellcome Centre for Cell-Matrix Research) for contributions to initial planning discussions.*

Author contributions: P.T. and R.L. compiled the article and P.T. prepared the figures.

### Financial support and sponsorship

The work was supported by a Wellcome Senior Fellowship award (202860/Z/16/Z) to R.L. and a Research Project Award to R.L. from Kidney Research UK (K1199). The authors also acknowledge core funding from the Wellcome Trust (203128/Z/16/Z) to the Wellcome Centre for Cell-Matrix Research at the University of Manchester.

### Conflicts of interest

There are no conflicts of interest.

## REFERENCES AND RECOMMENDED READING

Papers of particular interest, published within the annual period of review, have been highlighted as:

▪ of special interest
▪▪ of outstanding interest

## Figures and Tables

**Table 1:** Regulators used in six recently published kidney organoid differentiation protocols

Regulator	Differentiation direction	Differentiation pathway	Reference
CHIR99021 (Wnt agonist)	Primitive streak induction	Canonical Wnt signalling	[10,12,13^▪▪^,^14^^▪▪^,^15^,^19^]
Activin A	Primitive streak induction	Activin/BMP signalling	[12,15]
BMP4	Primitive streak induction Intermediate mesoderm induction	Activin/BMP signalling	[12,15]
FGF9	Intermediate mesoderm induction	FGF signalling	[10,12,14^▪▪^,^15^]
Retinoic acid	Intermediate mesoderm induction	Retinoic acid signalling	[15]

These protocols share some common features, for example, they all use CHIR99021 as a stimulator to induce the differentiation of human pluripotent stem cells from the pluripotent stage to the primitive streak stage. However, each differentiation protocol applies the regulators in a different order, combination, time, and concentration. BMP: bone morphogenetic protein; FGF9: fibroblast growth factor-9.
